# Continuous Attractors with Morphed/Correlated Maps

**DOI:** 10.1371/journal.pcbi.1000869

**Published:** 2010-08-05

**Authors:** Sandro Romani, Misha Tsodyks

**Affiliations:** Department of Neurobiology, Weizmann Institute of Science, Rehovot, Israel; University College London, United Kingdom

## Abstract

Continuous attractor networks are used to model the storage and representation of analog quantities, such as position of a visual stimulus. The storage of multiple continuous attractors in the same network has previously been studied in the context of self-position coding. Several uncorrelated maps of environments are stored in the synaptic connections, and a position in a given environment is represented by a localized pattern of neural activity in the corresponding map, driven by a spatially tuned input. Here we analyze networks storing a pair of correlated maps, or a morph sequence between two uncorrelated maps. We find a novel state in which the network activity is simultaneously localized in both maps. In this state, a fixed cue presented to the network does not determine uniquely the location of the bump, i.e. the response is unreliable, with neurons not always responding when their preferred input is present. When the tuned input varies smoothly in time, the neuronal responses become reliable and selective for the environment: the subset of neurons responsive to a moving input in one map changes almost completely in the other map. This form of remapping is a non-trivial transformation between the tuned input to the network and the resulting tuning curves of the neurons. The new state of the network could be related to the formation of direction selectivity in one-dimensional environments and hippocampal remapping. The applicability of the model is not confined to self-position representations; we show an instance of the network solving a simple delayed discrimination task.

## Author Summary

How is your position in an environment represented in the brain, and how does the representation distinguish between multiple environments? One of the proposed answers relies on continuous attractor neural networks. Consider the web page of your campus map as a network of pixels. Every pixel is a neuron, and nearby pixels excite each other, while distant pairs are inhibited. As a result of their interactions, a bunch of close-by pixels will light up, indicating your current position as suggested by your web-cam (the sensory input). When you travel to another campus, the common assumption holds that pixels are completely scrambled and the excitatory/inhibitory pattern of connections is summed to the existing one. Now these connections and the sensory input will activate the pixels corresponding to your location in the new campus. The active pixels will look like noise in the old map. But what if the campuses are similar, i.e. the pixels are not completely scrambled? We show that the network has a novel way of distinguishing between the environments, by lighting up distinct subsets of pixels for each campus. This emergent selectivity for the environment could be a mechanism underlying hippocampal remapping and directional selectivity of place cells in 1D environments.

## Introduction

The ability to keep an internal representation of a continuous variable in the absence of sensory stimuli, is a crucial requirement in order to succeed in what can be considered trivial day to day actions or experimenter designed tasks. For instance one may think about the eye position between successive saccades [1], the angle of stimulus presentation in an oculomotor delayed protocol [2], the spatial position or the head direction in a dark environment [3]–[5], or the phase of the recently discovered grid fields [6], [7].

A widely used class of models for this kind of working memory is constituted by attractor neural networks. The temporary maintenance of an item in memory corresponds to a specific network pattern of activity which is stabilized via strengthened recurrent connections between the active neurons in the pattern [8]–[11]. These connections are usually imposed, or trained, as the outcome of some form of Hebbian learning. The attractor is called continuous when the stable states form a continuous manifold which can be parametrized by the state variables. This outcome is obtained under certain conditions on the synaptic connection, for example when the connections between neurons are lateral-inhibition like (e.g. Mexican hat) [12]–[14]. The underlying idea is that each neuron is assigned a location on an abstract *map*. The synaptic weights (*encoding*) depend on the location of the pre- and post-synaptic neurons. By means of Turing instability, the network dynamics creates a localized pattern of activity (or bump) on the map [15]. The external input links the position on the map to the state variable, forming a *representation*.

Continuous attractors have been used to explain the maintenance of various analog quantities close or far from the primary sensory and motor regions. For instance, the orientation tuning in the visual cortex [16], [17], hippocampal place fields in one [18], [19] and two dimensions [20], [21], eye position [1], [22], head direction tuning in the postsubiculum [23], [24] and entorhinal grid fields [25], [26].

The simple picture of a single continuous attractor can be naturally extended to the case of multiple attractors. The encoded maps can then be assumed to be either uncorrelated or correlated, and in particular to exhibit some structure (e.g. deriving from a morphing procedure). Assuming a complete lack of correlations between maps is not realistic, though useful for obtaining analytic results [27]. In this contribution, we analyze the network representations arising from the storage of two maps, with a varying degree of correlation between them, and from the storage of a morph sequence between two uncorrelated maps. We are interested in finding the conditions under which the network representation can provide some information about the state variables. Surprisingly, even when the correlation between two maps is very high, under conditions which will be clarified later it is possible for the network to maintain separate representations of the state variables.

### Multiple maps

Multiple state variables can be encoded in the same network. An example is offered by the place representations of several environments [20], [21]. To each environment corresponds a neural map which is encoded in the synaptic efficacies. Sensory inputs would then select the correct representation, i.e. both the environment and the position in the environment. The selected map wins the competition with the other maps stored in the network, and a localized pattern appears. In this case the network only maintains information about one of the several encoded state variables.

A more peculiar property of multiple continuous attractors, is their ability to represent simultaneously the values of several state variables. This property was explored in [28], where two partially overlapping neural populations (representing discrete features), are assigned two uncorrelated maps. Another example is provided in the study of [29], where a single network stores and represents simultaneously a continuous and discrete attractors.

In principle, given the existence of multiple representations in different brain regions (either one per region, or many in one region), a brain area downstream would necessarily encode several state variables. In light of a Hebbian interpretation on how this encoding takes place, it seems natural to distinguish between two cases. When multiple representations provide a simultaneous input to a region, the result is probably encoded multiplicatively [29], or, in general, non-linearly. For inputs happening non concurrently, as for instance when walking through several rooms sequentially, an additive encoding of each room is expected [21]. In the following we will analyze additive encoding.

### Correlations

The present contribution addresses the issue of encoding correlated maps. The motivations come from recent experimental results on place cells recording in morphed environments [30]–[32], where place fields remapping along a sequence of morphed arenas is experimentally tested, and from theoretical and experimental studies concerning the morphing of discrete attractors [33]–[35].

In general, we would consider the encoding of 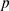 manifolds 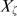, each of dimension 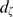, where 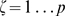. We will refer to a single manifold as a map, once a coordinate system 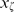 is chosen. The use of uppercase (e.g. 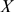) or lowercase (e.g. 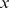) will distinguish between the whole map and a single point on it respectively. Given a pre-synaptic neuron indexed by 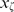, and a post-synaptic 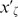, the encoding of a single map is obtained using a synaptic matrix 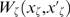, and is such that a continuous attractor representation would arise if it were the only map. We assume, as mentioned above, that the complete encoding arises from a linear superposition of the 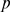 matrices, 
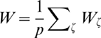
. The statistical properties of the maps, and in particular the correlation between them, can be fully specified by providing the probability density 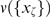.

The general problem is too difficult to be studied analytically. Some results can be obtained for the case of uncorrelated maps on the same manifold [27], though the system can be explored by simulating the full microscopic networks (see e.g. [21] for the uncorrelated case and [36] for simulation results of the correlated case).

In order to simplify the analysis, while retaining the basic structure of the problem, we focus on the case of 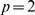 representations, on a 1-dimensional circular manifold (i.e. the ring model [16], [37]). The correlation between the maps is constructed by limiting the distance between the single neuron locations on the two maps. We devise a simple method to generate a morph sequence between two uncorrelated maps, by linearly modifying the neurons locations between the original maps. This method also suggests a way to test the network response to the exposure of intermediate maps between the two stored correlated maps.

For concreteness, one could think about maps of two similar circular arenas, and reason in term of spatial coding. In this context, we are interested in clarifying how the information about the position in the current environment is represented by the network, when varying the constitutive parameters of the model; And how the representation changes when the network is exposed to environments along a morph sequence.

In the following we will describe with mean-field (MF) theory the attractor landscape of a network, i.e. the stable solutions in absence of any place specific input. We then consider the behavior of the solutions when a spatially tuned input is present. We will establish the approximate relationship between two strongly correlated maps and the encoding of a morph sequence between two reference rings, and study the behavior of the solutions in presence of a tuned input varying along the sequence. Finally we will verify the results with microscopic simulations of finite networks. The network properties can be tested experimentally to confirm (or falsify) the attractor hypothesis.

## Results

Let us consider two circular environments 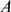 and 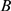, inducing two different maps 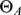 and 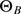 in the network. In the MF limit, we can imagine having a continuous manifold of neurons, where each neuron is identified by the pair of labels 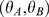, with 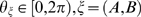. In addition, a probability density 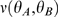 tells us how likely is for a neuron to have the labels 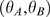. As mentioned in *Introduction*, we assume the resulting synaptic structure to be a linear superposition of ring models. Hence, the connection strength between two neurons 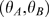 and 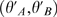 is
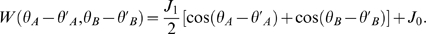
The factor 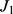 is a measure of the amplitude of the map specific interaction, while 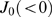 is a uniform inhibitory term. This form of connectivity can be thought as arising from the first two terms of Fourier series of a more general coupling. The rate dynamics for the network activity 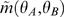 is [38]:
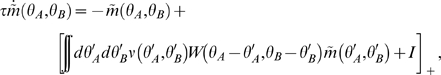
where we assumed a threshold-linear transfer function for the neurons, 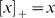 when 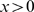 and 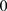 otherwise. The external afferent current is denoted by 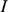, and it is assumed uniform in the current Section.

**Figure 1:**
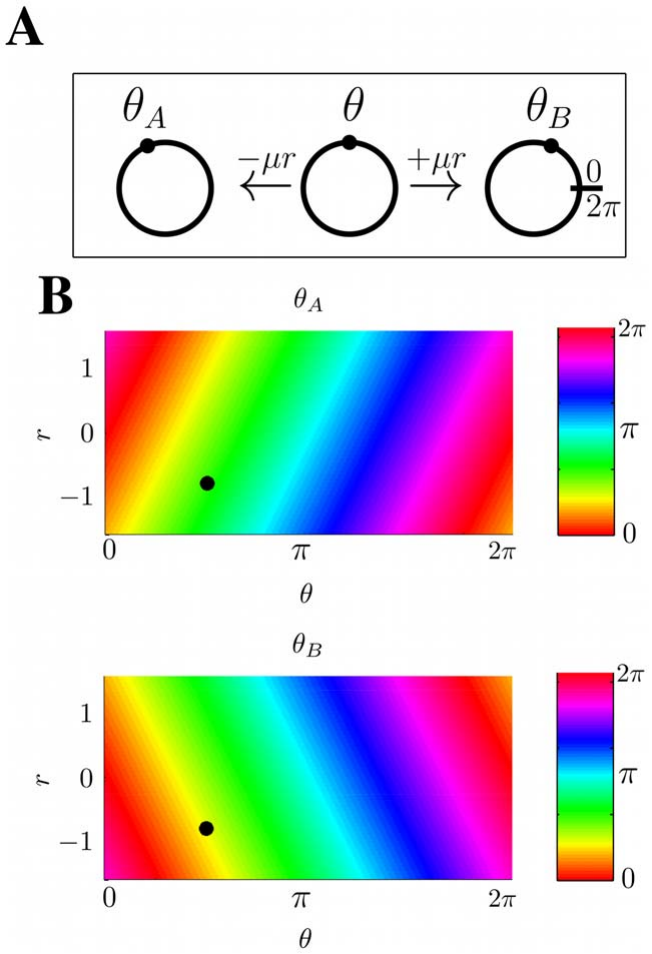
Construction of correlated maps. **A:** Cartoon showing how to generate two correlated maps 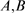 from neurons with randomly assigned index 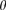 on a reference ring, and distance value 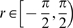. Given the distance between maps 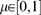 (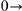
identical, 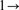
uncorrelated), the desired maps are created by adding and subtracting the value 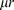 to the reference location 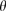. The reference acts as an intermediate map, and the distance between maps limits the maximum distance between the location of the neurons on the maps 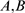. **B:** Color codes corresponds to angles in map 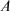 (top panel) and 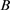 (bottom panel). The two plots show how neurons indexed by 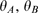 are represented in 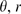 coordinates, for 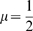. The particular neuron depicted in the cartoon is represented as a dot.

We build the maps with a simple procedure which induces a correlation between them. First, we create a uniformly distributed map 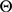 with coordinates 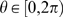 and a uniformly distributed map 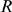 of distance values 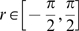. Then we define the coordinates of the desired maps as
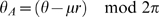
(1)

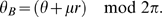
The parameter 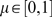 is a measure of the distance between the two maps; the higher the distance between the maps, the lower the correlation between them. The coordinate 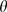 defines a “middle” map from which the coordinates of the environments 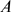 and 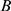 are constructed; each of them cannot be farther than 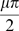 from the middle map, hence they cannot differ more than 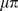. When 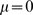 the two maps are identical, and for 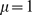 the two maps are uncorrelated. As an example, let us fix the distance between the maps at 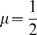 and consider the case of a neuron with 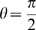; a choice of 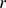 for this neuron will yield the coordinates in the maps 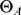 and 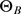. The range of possible values for 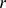 will generate 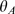 and 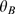 in the interval 
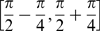
, which shows how not all the possible pairs 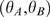 are obtainable. An instance of this procedure is depicted in Fig. 1. A given angle in map 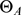 or 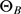 (corresponding to a given color in Fig. 1**B**) is represented by a straight line in the reference frame 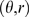. The effect of a decreasing 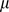 is to tilt this straight line toward the vertical direction (only identical angles in map 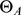 and 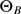 would be possible). Note that it is possible to define the inverse transformation 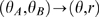 (Eq. 21).

The new coordinates 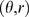 are uniformly distributed by construction. We can then rewrite the dynamics of the network activity 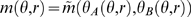, using Eq. 1, as
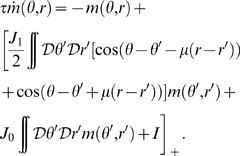
(2)The notations 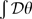 and 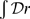 are a shorthand for 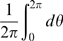 and 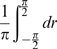 respectively. The use of the ring connectivity structure makes possible to reduce the dimensionality of the dynamical system to few *order parameters*. Five order parameters are necessary in order to describe the dynamics of the system: 
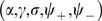
 (see *Methods* *- Reduced Dynamics* for the details of the derivation, and the next Section for the equations describing their dynamics). Our choice of the order parameters exclude the analysis of the uniform solution of Eq. 2, i.e. a constant activity over the whole network. We will return to this solution in *Results* *- Phase diagram of the model*. After the reduction, the steady state activity profile in 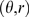 space assumes the form:
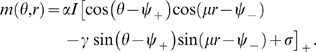
(3)Note that a change in the strength of the applied uniform input 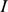 produce no changes in the order parameters (see *Methods* *- Reduced dynamics* and Eqs. 5).

**Figure 2:**
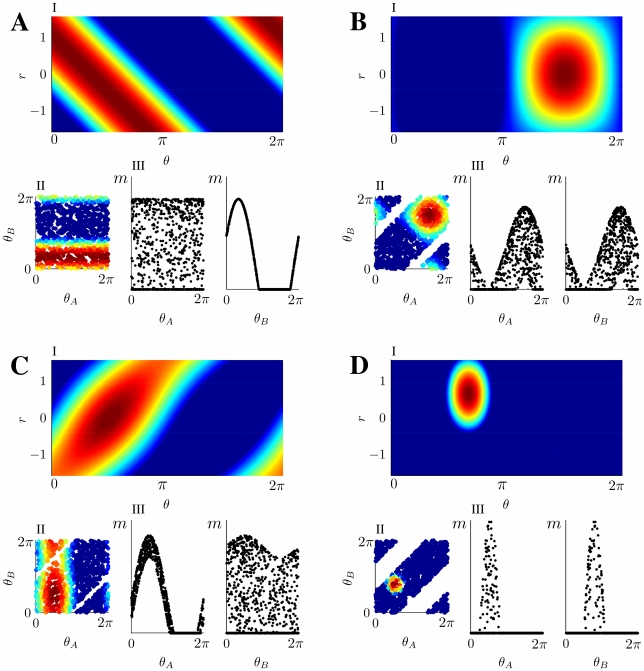
Network activity examples. The top plots (numbered 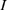) in each panel show the MF network activity (Eq. 3) in the 2D map 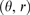, corresponding to different choices of the order parameters 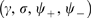 and the distance 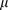. Each point in the plot corresponds to a neuron with labels 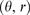, the color is proportional to the activity level; warmer color represent higher activity, blue represents no activity. The activity scale is arbitrary, since it can be rescaled by a change in the external input 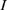, without modifying the shape of the bump. Plots on the left of each panel (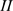) show the corresponding activity for a network of 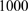 randomly chosen neurons in the 2D map 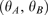, see Eq. 1. The white stripes in the plots are due to the absence of neurons with labels in those regions, imposed by the correlation between the maps. The bottom-right pairs of plots (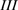) are projections of the activity on the individual maps. **A:** 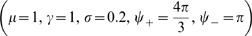 Activity localized in a single map, from a network storing uncorrelated maps. The locations of the peak activity in map 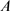 and 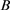 are 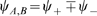. **B:** 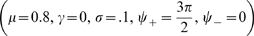 Correlated maps, activity not favoring either 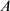 or 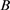; since 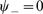, the bump is located at the same angle in both maps. **C:** 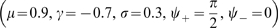 Correlated maps, activity prefers map 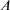 (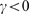). **D:** 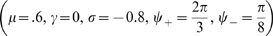 Correlated maps; for this smaller sized bump, the location of the activity maximum is such that 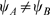.

Several examples of network activity (Eq. 3), corresponding to different representative choices of the order parameters, are shown in Fig. 2. The various panels show the network activity in the two two-dimensional maps 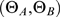 and 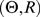, and the one-dimensional projections of the activity to 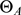 and 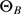. Note that not all the choices of order parameters corresponds to actual solutions of the dynamics (which are determined by the parameters 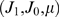 and the initial conditions), as will be shown later.

The meaning of the order parameters can be read out from Eq. 3. The variable 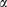 represents a scaling factor for the amplitude of the network activity, which in turn is proportional to the uniform input 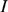.

The variable 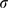 is a measure of the spatial size of the activity profile, i.e. of the region in either 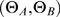 or 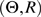 in which the network activity 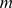 is strictly positive (Eq. 3). The activity profile is also referred to as a bump. For instance the case 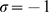 would correspond to absence of activity (the current in the threshold-linear transfer function would always be negative), while 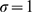 would make all the neurons in the network active.

The order parameter 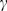 tells us how much the network representation “favors” one of the two maps. By its definition (*Methods* *- Reduced dynamics*, Eq. 24), the possible range for 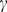 is 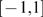. The two extreme cases 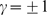 correspond to a network activity localized in either map 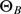 or 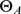. For instance, the network activity Eq. 3 for 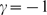 reads
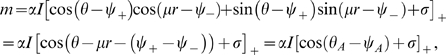
where in the last equality we used Eq. (1). From here we see that the position of the bump peak is located at 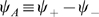; the same derivation, with 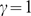, would give us 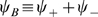. The network representation is in this case a bump of activity localized in one map, and does not have any spatial modulation in the other map, as exemplified in Fig. 2**A,III**. In the case 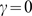, from the explicit expression of the activity 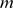 we get
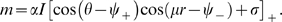
(4)This representation exhibits an equal amount of spatial modulation in both maps 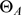 and 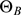, i.e. the solution represents equally the two stored maps, Fig. 2**B,D**. Depending on the value of 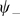 (see below), the location of the bumps in the maps 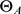 and 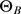 can be either the same (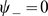, Fig. 2**B,III**), or different (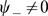, Fig. 2**D,III**). Solutions with intermediate 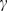 values (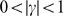) have a more localized projection in one of the two maps, depending on the sign of 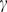 (see for instance Fig. 2**C**).

The quantities 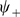 and 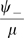 identify respectively the location of the maximum of the network activity in the 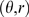 coordinates, which is uniquely mapped to the maximum in 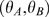 via Eqs. 1.

In the following, we will show that the network activity examples depicted in Fig. 2 are possible solutions of the dynamics described by Eq. 2. We refer to each of these classes as *double ring* (Fig. 2**A,C**,) *single ring* (Fig. 2**B**) and *cylinder* (Fig. 2**D**), for reasons that will be clarified in the next Section. The cylinder class represents an interesting novel regime (simultaneous localized projections in both environments), and we will devote most of the paper to describe the properties of this particular solution.

### Phase diagram of the model

In this Section we analyze the fixed point solutions of the system, and heuristically describe the region of stability of these solutions. A more rigorous description of the stability can be found in *Methods* *- Stability*.

In *Methods* *- Reduced dynamics* we derive the dynamics of the order parameters from Eqs. 2. We report here the result
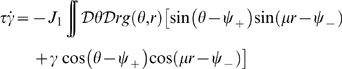
(5)

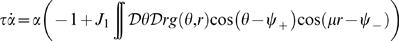


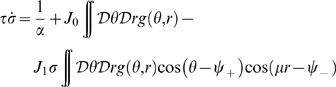


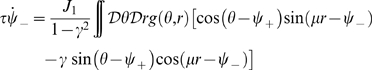


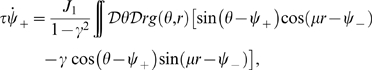
where the function 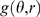 is defined as
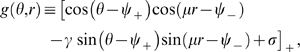
(6)i.e. the rescaled steady state activity profile Eq. 3. Note that 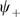 can be eliminated from the right hand sides of the Eqs. 5, rotating the integration variable 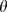. This is possible because there is no spatial dependence in the external input to the network. The first four equations in Eqs. 5 can then be solved independently of the fifth one, since the right hand sides do not depend on 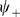. We show in *Methods* *- Solutions properties* that, once we have the solution for the variables (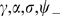), the last equation reduces to 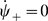. We can thus restrict the analysis to four out of five equations in Eqs. 5. The elimination of one angular degree of freedom is a consequence of the rotation invariant structure of the encoding, and is the hallmark of continuous attractors arising from spontaneous symmetry breaking. On the other hand, the integrals over 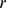 in Eqs. 5 are not over the whole circle and we cannot rotate 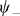 away.

### Homogeneous solution

**Figure 3:**
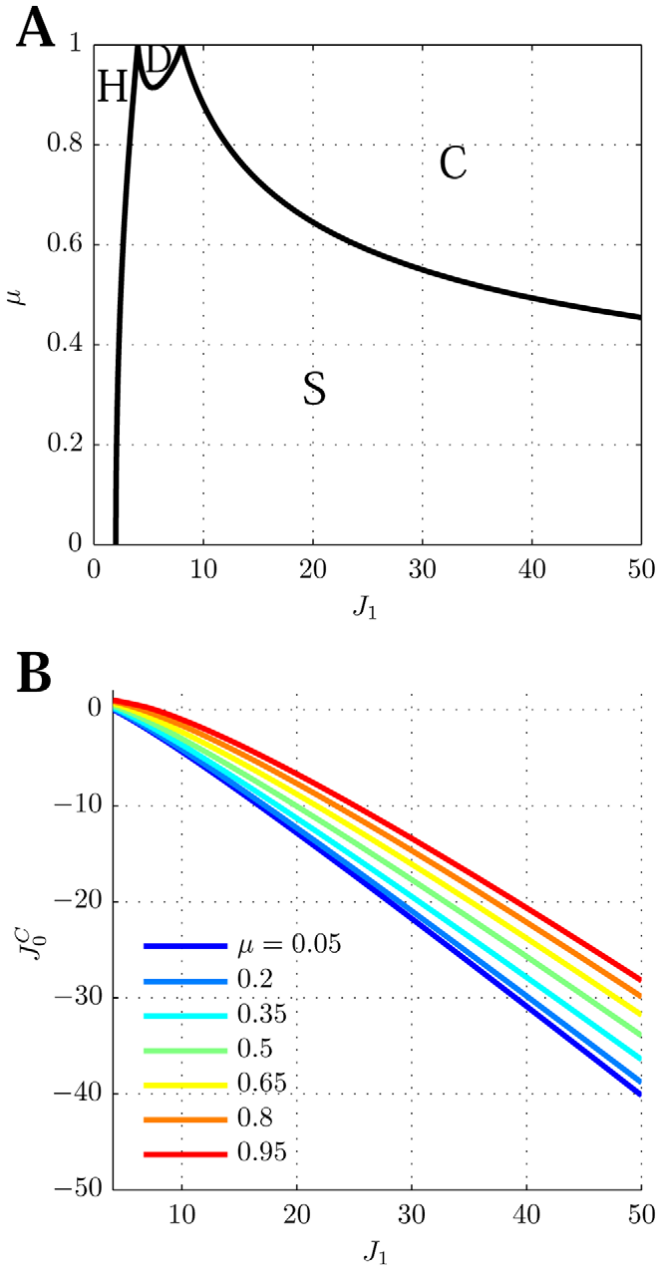
Phase diagram. **A:** MF solutions in a network storing two correlated maps. The black curves in the 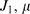 parameter space depict the separation between the qualitatively different solutions of the system. *Homogeneous* solution (denoted by H in the panel), all the neurons are active at a constant level. *Single Ring* (S) solution, localized activity in the middle map, the bump can be freely rotated in this map. *Double Ring* (D) solution, pair of solutions localized either in map 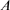 or 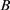. *Cylinder* (C) solution, the network activity is localized in both maps; compared to the single and double ring solutions, there is an additional freedom in the choice of the location of the bump over 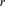 (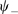). The term cylinder is used because the continuous attractor lives in the space defined by the angle 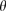 and a sub-segment of 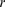 (with the exception of 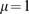, see main text for details). **B:** Amplitude instability. Critical inhibition 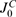 corresponding to the onset of unstable solutions for varying 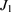. Each curve corresponds to different distances between the maps 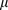.

Before analyzing the fixed point solutions of the system described by Eqs. 5, we briefly mention an uninteresting region in the parameters space which can be found also in the classical ring model. This region corresponds to the homogeneous solution, i.e. all the neurons in the network are active at a constant level, and can be obtained from Eq. 2. The expression corresponding to the line of separation in the plane 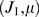 between the homogeneous solution and the spatially localized bump (see Fig. 3**A**, curve surrounding the **H**omogeneous region), is
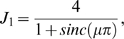
(7)where 
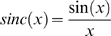
. This result is obtained in *Methods* *- Stability*, see also below.

### Fixed point equations for the localized activity state

Let us start by imposing 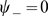, a restriction that will be addressed later on. The first tree equations at steady state from Eqs. 5 become then equations for the three order parameters 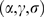:
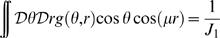
(8)

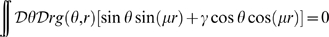


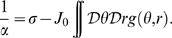
The first two equations determine the shape of the bump 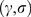. Given the map specific modulation in the coupling and the distance between the maps 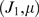, we can derive from the first two equations the size of the bump 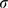 and the order parameter 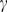, representing how close the network representations are to the stored environments 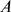 and 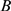. The last equation gives us the amplitude of the network activity 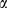, which also depends on the parameter 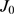.

As mentioned in *Results* *- Phase diagram of the model*, the order parameter 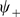 can be chosen arbitrarily, due to the rotation invariance of the problem; for simplicity we choose 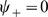.

### Amplitude instability

We deal first with the equation concerning the amplitude of the solution. Given that the activity can be rescaled by changing the value of the applied external current 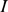, we are not interested in actually solving the equation. The only requirement is that 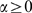 in order for the solution to be meaningful, i.e. no negative amplitudes are allowed. This requirement translates to a constraint on the inhibition 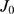:
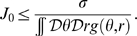
(9)We show with stability analysis (*Methods* *- Stability*) that the critical value 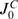, obtained by choosing the equality in the previous expression, corresponds to the onset of amplitude instability; given a choice for the parameters 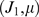, which specifies the bump shape 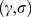, for values of the inhibition weaker than 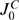 the solution grows to infinity. This qualitative behavior was present also in the classical ring model.

Fig. 3**B** shows the values of 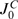 as a function of 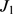 for various choices of 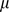. In order to stabilize the solutions, the inhibition must grow with increasing 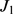 and decreasing 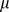. Note that it is reasonable to consider the previously mentioned homogeneous solution as a bump with maximal size 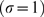. In this case the critical 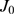 can be explicitly computed, and turns out to be 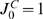.

### Single ring solution

Now we focus on the possible solution 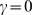. It is easy to see that when 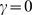, the second of Eqs. 8 is automatically satisfied due to the symmetry of the integrand in 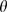 (and 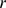); This means that the solution 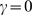 exists everywhere in the parameter space.

The steady state activity Eq. 3 with 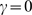 (and 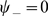, our initial assumption) reads
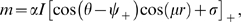
(10)which corresponds to a packet of activity localized in the 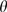 coordinate, and modulated in 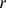, see Fig. 2**B** for a plot of the activity profile. The remaining fixed point equation can be used to obtain 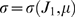. We refer to the case 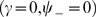 as a single ring solution; the ring is spanned by the freedom of choice in the angle 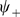. In this regime of activity the network is not able to represent separately the environments 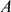 and 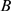, but only the middle environment described by 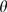. Even though the solution exists everywhere, it is destabilized in some regions of the parameter space, as shown in the phase diagram (Fig. 3**A**, **S**ingle ring region).

By looking at the maximal bump size 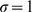, we can expect to reproduce the curve separating the homogeneous solution from the single ring. Inserting 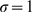 in the first of Eqs. 8, it is possible in this case to compute explicitly the integral, which in fact yields Eq. 7.

### Double ring solution

**Figure 4:**
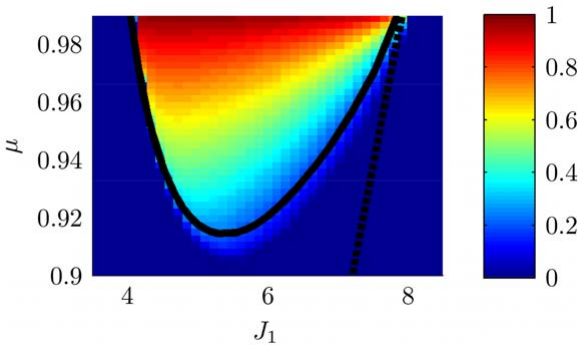
Double ring solutions. Region in the parameter space 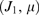, where the double ring solution 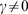 exists. The color correspond to the value of 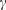 which solve Eqs. 8. Due to the symmetry of equations, both positive and negative 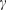 values are allowed. Here only the positive solution is shown, corresponding to a localized solution in map 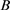. **Black curve:** curve of separation between the null and positive 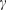 solutions, obtained by finding the zeros of Eq. 12. Note that the method used to obtain the curve is more precise than the one used to estimate 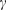. **Dashed curve:** curve corresponding to a network activity size 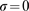. Regions on the left of this curve have 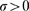, which is a limit size of the double ring solutions (reached at 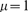).

In order to find the region of existence of the solutions with 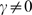, we can solve numerically Eqs. 8 in the parameters plane 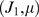. The result is shown in Fig. 4, where the color code represents 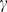 for a given choice of the parameters. It can be seen that there is only a narrow region of high 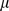 (low correlation) and low 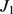 where such a solution exists.

It is important to note that the equations used to find 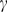 are invariant under the symmetry 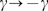. This means that both solutions (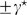) representing map 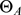 or 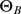 are possible. The steady state activity profile in this case looks like:
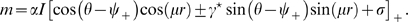
(11)Given the freedom of choice for the phase 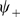, each of this solutions lives on a ring; we call the solution 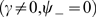, double ring. An instance of the network activity in this regime is shown in Fig. 2**C**.

The curve separating representations preferring one of the two maps (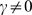), and 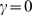, can be obtained by expanding the second of Eqs. 8 to first order in 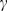:
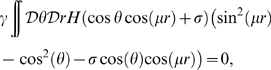
(12)where 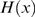 is the Heaviside step function, 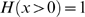 and 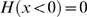. Dividing by 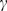, we get rid of the 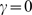 solution. By finding the zeros of the integral, we select the curve in the parameter space corresponding to the onset of existence of the double ring solution. This curve is shown in Fig. 4. We have found that the stability of the double ring solution coincides, empirically, with the region of existence of such solution (compare the phase diagram in Fig. 3**A**, **D**ouble ring region with Fig. 4).

### Cylinder solution

Finally, we examine the meaning of the equation for 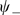, the order parameter linked to the location of the maximum of the bump in 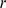. We have assumed 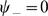 for simplicity, given that a rotation in the integrands in Eqs. 5 is in general not viable due to the restricted range of integration in 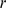. Note though, that when the size of the bump 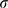 is small enough, it is possible to perform the rotation without affecting the value of the integrals; the only requirement is that the rotation keeps the bump from touching the boundaries 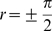.

In *Methods* *- Solutions properties* we verify that there are no solutions with both 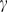 and 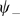 different from 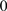. We can therefore set 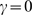 in the steady state activity Eq. 3, and impose the activity itself to be zero on the boundary 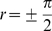 to find
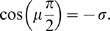
This equation corresponds to the curve of separation in the plane 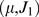 (using the relationship 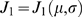, Eq. 8) between the single ring solution and a *cylinder* solution (Fig. 3**A**, curve surrounding the **C** region). In this regime, in addition to the freedom of choice for the location of the bump in 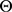, the solution is also partially marginal in 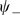. The bump can be freely moved on a segment and a circle, defining a cylinder; the activity profile in this case is described by Eq. 4, see an instance in Fig. 2**D**. This region extends in the high 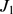 limit and covers the whole range of correlations.

Despite the fact that each of the maps 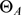 and 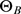 defines a ring, it shouldn't come as a surprise that the topology of the attractor is a cylinder instead of a torus. The correlation between maps gives rise by definition to a cylinder structure, as can be seen for instance by inspecting Fig. 2**B,II**. It can be shown that when 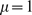 the cylinder solution degenerates in a torus; the bump of activity can be in any location of the 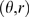 coordinates (hence, also in 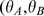)). This regime is linked to the observation of an activity bump simultaneously localized in two environments in network simulations [39], and the study in [28].

### Phase diagram

Fig. 3 summarizes the results obtained so far. When 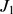 is low, the only solutions is a constant level of activity which spreads over the whole network (**H**omogeneous region). As 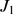 is increased, the interplay between the short range excitation and long range inhibition creates a pattern of localized activity in the middle map 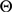 (**S**ingle ring, see also Fig. 2B) or, if the correlation between maps is small enough, a localized pattern in either 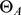 or 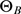 (**D**ouble ring, Fig. 2C). Intuitively, the network “remembers” the two maps separately (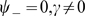, two solutions) if they are weakly correlated (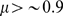). When the maps are more similar, the network represents just an average between them (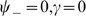).

The bump size decreases with increasing 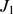. When 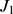 is further increased, instead of having a reduced size of the localized activity in just one of the maps, the presence of two stored maps in the synaptic structure and the inhibition 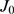 produce a packet of activity which looks localized in both maps (**C**ylinder solution, Fig. 2D).

Three particular values of the distance 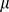 deserve a special mention. The case 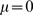, corresponding to the encoding of two identical maps, can be shown to be identical to the ring model [37], as expected. In particular, besides the homogeneous solution and the amplitude instability region, the system can only exhibit the single ring solution.

The case 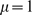, corresponding to the encoding of two uncorrelated maps, does not have the single ring regime as a possible solution. The double ring solution in this case is depicted in Fig. 2**A**, where it can be seen that the bump is perfectly localized in either maps 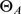 or 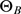, lacking any spatial tuning in the other map. This is the desired outcome in the “multi-chart” approach of [21].

The third case is 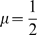. We will see in *Results* *- Morphing maps* that this case is closely related to the behavior of a network storing a morph sequence between two uncorrelated maps. As can be seen in the phase diagram, the double ring solution is not possible in this regime.

How the environment, and the position in the environment, are represented by the network activity? For the single ring (Eq. 10) and the double ring (Eq. 11) solutions, both characterized by 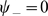, it is evident that the position is coded by the order parameter 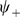. The identity of the environment can only be represented with the ambiguity in the choice of the sign of 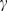 when the network operates in the double ring regime.

In the cylinder regime, it is not clear how the information about the environment is represented in the network, since now the solution is described by 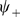 and 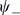. The following Section is mainly devoted to explore the link between the state variable (eventually time-dependent) 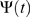 in the active environment, and the behavior of the solution in this novel regime, by introducing a spatially tuned external input.

### Tuned external input

Until now we considered the condition in which the only external input to the network, 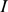, was steady and uniform. Let us introduce a tuned input, for instance in map 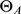 at position 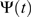:
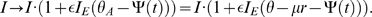
For simplicity we assume the shape of the external input to be 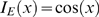. The parameter 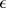 measures the strength of the tuned component of the external input as a fraction of the constant baseline 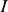 we adopted so far. In general what we are interested in, and what is experimentally observable, are the tuning curves of the neurons i.e. their profile of activity as a function of the input angle in the active environment. It is easy to see the effect on the dynamics of the order parameters (Eqs. 5) when the location specific external current is inserted in the original dynamics for the network activity, Eq. 2. The dynamics keeps the same form as in Eq. 5, with the exception of the threshold-linear term in 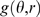, which now reads
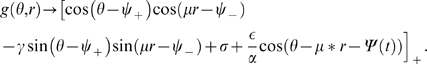
(13)where 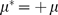 correspond to the choice of map 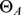 in the input, and 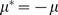 for map 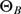.

### Tuning curves

With the input at a constant location 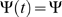, one can see that a solution of Eqs. 5 for the single and double ring regime (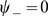), is 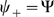, i.e. the input pinpoints the location of the bump. This implies that, assuming a weak tuned input 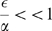, the tuning curve of a neuron 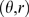 can be written in the single and double ring regime (from Eqs. 10,11) as
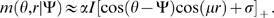
(14)and
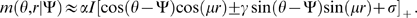
(15)respectively. The tuning curve in the single ring regime has a maximum for 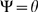 (hence 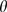 is the preferred angle for a neuron 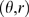), independently of which map 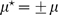 is being used in the external input, as can be seen from Eq. 14. This implies that each neuron has identical tuning curves in both environments, and that the preferred angle of a neuron does not coincide with either the assigned 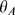 or 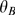 but with their average.

For the double ring regime, the preferred angle assumes the form (maximizing Eq. 15 in 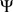)
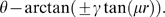
In this case each neuron has two different tuning curves according to the map used in the external input. The preferred angles coincide with the assigned ones (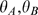) only when the stored maps are uncorrelated (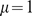, hence 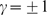).

In the cylinder regime (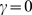, 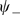 not necessarily 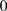), a solution for Eqs. 5 in presence of a tuned input is 
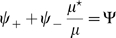
. For an input in map 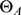, 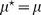, the tuning curve would then be proportional to (from Eq. 4)
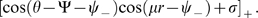
Note that the dependence on 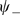 means that the external stimulus does not determine completely the network activity, in contrast to what happens in the previously examined regimes. Neurons that respond maximally to the tuned input are then 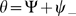, and 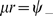, hence 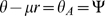. This means that the tuned external input pinpoints the location of the bump maximum in map 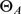 but the bump is free to stabilize anywhere along the other map given the freedom of choice in 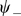 (see activity example in Fig. 2**D**). If several randomly selected external locations 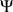 in one of the maps are presented to the network, once at time and starting from random initial conditions, the tuning curves would be an average over 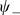:
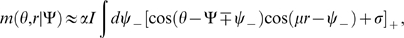
where the allowed range for 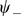 is 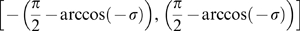, see *Methods* *- Solutions properties*. The cylinder regime extends the region of existence of two tuning curves per neurons to an higher correlation between the stored maps; the difference is that the coding becomes *unreliable*: during a single exposure to a given value of the input angle 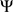, a neuron could remain silent even if its average tuning curve would predict a response.

When the representation refers to the location in an environment, it is natural to think about a smoothly varying location 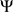. With a moving input like 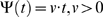, the tuning curve depends as before on which map is stimulated, but in a novel way. Assume for simplicity to start from a 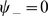 initial condition, corresponding to (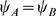). A moving input in the map 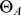 would tend to move the bump along that map (i.e. increase the 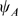 of the solution), while keeping 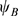 constant (hence the bump will move to 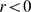). This movement is possible only until the bump reaches the part of configuration space not occupied by neurons due to the distance between maps 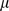, see Fig. 2**D**. At that point, the bump will start to move equally along 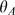 and 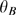, maintaining 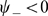, which is proportional to 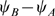, and increasing 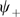 (proportional to 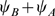). A similar scenario, but with 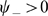, is obtained when stimulating the map 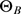.

**Figure 5:**
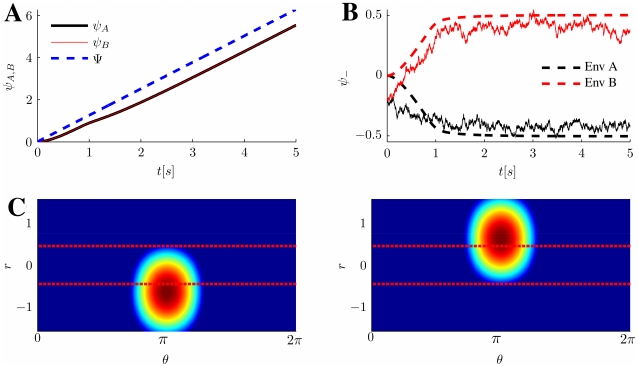
Network representations in the cylinder regime. **A:** MF time-course of 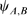, when a moving input localized around 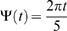 in either map 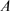 or 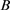, is fed to the network. The location of the maximum of the external input in the stimulated map is shown with a dashed blue line. The order parameter 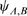 tracks the external input with a delay. **B:** The order parameter 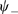, giving the position of the bump in 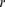, with the same moving inputs. Depending on the stimulated map, the bump ends up in different positions, orthogonalizing the representations. Noisy curves from a simulation including a zero average white noise term in the external input, with 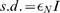. **C:** A snapshot of the network activity at a given time 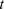 and corresponding angle 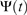, when the stimulated map is 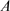, left or 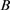, right. Neurons with 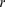 values differing enough from 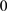 (depending on the size of the bump) will exhibit tuning curves only when stimulated in one of the two environments (above/below red dashed lines). The parameters for all the panels are 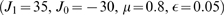.

If the size of the bump is sufficiently small, this effect has dramatic consequences. The small bump will move along neurons with 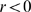 when a moving stimulus is presented in environment 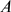, and viceversa neurons with 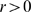 will be active only when the moving stimulus is presented in environment 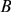. As a consequence, neurons will essentially just have a tuning curve (or field), only in one map, and will be silent in the other one. We refer to this phenomenon as dynamical pattern separation (see Fig. 5 for an example). The separation of the activity patterns is essentially a dynamical phenomenon, dependent on the history of the inputs. The figure shows also the robustness of the dynamical pattern separation behavior to the addition of Gaussian 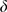-correlated noise in the external current (see *Methods* *- Numerical Methods*). Note that neurons characterized by 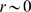 (i.e. 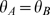), will have tuning curves in the same location. The number of neurons with tuning curves in both environments grows with the size of the bump.

Note though that by changing the sign of the velocity in the moving input, the behavior would reverse; neurons with positive (negative) 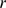 would be active during a stimulation in map 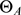 (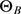). In order to maintain the dynamical pattern separation and the analogy with place coding, one could think about two circular environments, as we did so far, with the additional constraint that the environments can only be traveled, for instance, in the counter-clockwise direction (CCW). As an alternative, the two environments may be thought as the same circular arena, but traveled clockwise (CW, environment 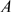) and CCW (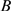); this interpretation would give rise to place fields with directional selectivity (see Discussion).

The dynamical pattern separation is basically dependent on the history of the input (positive or negative velocity), in addition to the identity of the map used in the stimulation. This history dependence is present also for non smooth time-dependent stimuli, as for instance the sequential presentation of stimuli with an intervening delay period. In this case the history dependence gives rise to a memory effect: the current location of the bump following a stimulation depends on the location attained after the previous stimulus presentation. Let us consider a basic example of this phenomenon, where the tuned external input is always presented in map 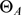. Consider for simplicity the state of the network being characterized by 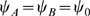, as a result of the presentation of stimulus 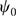 sometime in the past. If we now present a stimulus 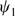, the bump will move, through the shortest arc on the map, to the new location 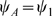. Depending on the stimuli, this movement can happen in two ways. If the shortest arc from 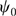 to 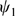 is directed CCW, the bump will move with a positive velocity 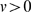 and will end up being located in the 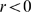 region (as we previously saw in the case of moving tuned input). If the shortest arc is directed CW, then the movement will happen with a negative velocity, and the final location of the bump will be in the 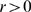 region. Hence, by looking at the activity resulting from the presentation of 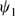, we know whether the shortest way on the ring to it from 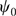 is CW or CCW. A similar result can be obtained if the stimulus presentation alternates between map 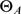 and 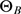.

If we vary the manifolds on which the maps live, for example to segments instead of circles, the history dependence changes accordingly. For instance, on segments the activity would give us information about the second stimulus being greater/smaller than the first one (see Discussion). In the next section we present a simple (albeit artificial) delayed discrimination task which the network can perform by exploiting the memory effect.

### An application of the memory effect

Let us suppose to have a screen with a circle on it. A first stimulus (a dot) appears on the circle at some random location (described by an angle, 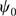), for the duration of 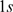. This first stimulus is then removed for a delay period of 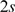. Then a second stimulus appears at another random angle 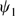; the subject's task is to determine whether the shortest path on the circle from angle 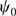 to 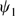 is CW or CCW. The basic idea is that it is enough to look at the network activity (location of the bump in the 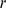 axis), to determine the relationship between the first and the second stimulus (see *Results* *- Tuned external input* for a description of the idea).

**Figure 6:**
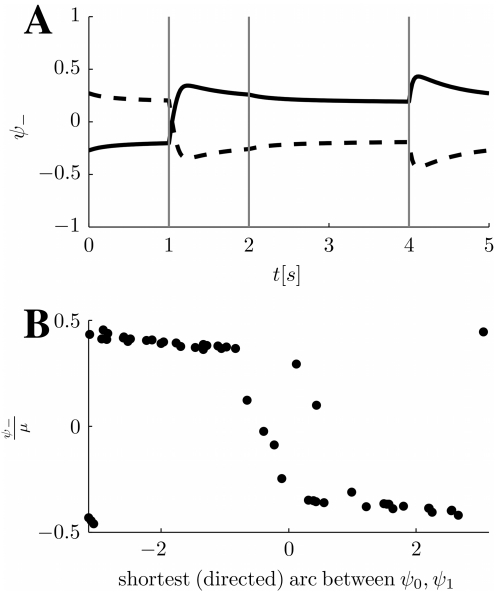
Solving the shortest path task. **A:** Two instances of MF dynamics from a network storing two correlated maps, during the presentation of two angles (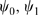) with an intervening delay. The network operates in the cylinder regime. The plot shows the time course of the order parameter 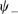; 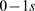: pre-stimulus period, random starting 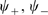, no tuned input. 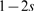: first stimulus (presented in map 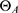, at 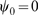 (solid and dashed). 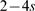: delay period, no tuned input. 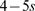: second stimulus 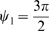 (solid) and 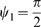 (dashed). By looking at the position of the bump in 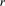 (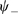) during the second stimulus presentation, it is possible to decide whether the shortest path between the first and second stimulus is CW (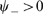) or CCW (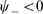). **B:** Sequential repetition of the task, 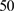 trials. The location of the bump maximum along 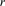 is plotted 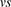 the oriented distance on the circle between the second and the first stimulus. Parameters 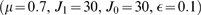.

To test the ability of the network to solve this task, we numerically solve the dynamics for the order parameter (*Results* *- Phase diagram of the model*) with an external input (*Results* *- Tuned external input*) mimicking the presentation of the stimuli, for a sequence of 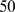 trials. We used no inter-trial interval, i.e. the presentation of the second stimulus in the 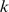-th trial is immediately followed by the presentation of the first stimulus in trial 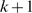. The time courses of the bump location on the 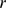 axis (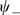) in two example trials for which 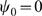, are shown in Fig. 6**A**. When looking at the location of the bump in the 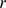 axis at the end of a trial, there is a clear difference between the two cases of shortest CW, corresponding to positive 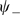 (in the specific example 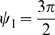), or CCW arcs (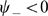, where 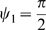). Fig. 6**B** shows that the bump location at the end of trial, can be used to easily discriminate between the two possible answers (except for the cases in which the first and second stimuli are relatively close to each other). Note that this result has been obtained without any activity reset to new initial conditions during the inter-trial intervals.

### Morphing maps

How do the results described so far change when, instead of storing just two correlated maps, the network encodes a sequence of maps gradually morphed between two uncorrelated ones? Let us start by constructing two random uncorrelated maps, 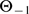 and 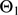. We would like to define the intermediate maps as gradual rotations between the two extreme ones; since we are dealing with circles, the rotation should be performed along the shortest arc between 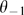 and 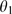 (see Eq. 21, *Methods* *- Inverse transformation*). We assume here to have already transformed the variables in such a way that we can write directly
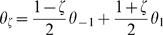
(16)where 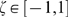 indexes the maps along the morph sequence. Hence a neuron with label 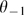 in the first map, will rotate along the sequence to its location 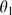 on the last map, following the shortest path on the circle. With this choice of the morphing procedure, each neuron is still characterized by just two quantities, its labels in the extreme maps.

We store the whole morph sequence by a superposition of the synaptic structures generated in each map separately, as for the case of two correlated maps previously described. For the sake of analytical tractability, we study the resulting coupling in the limit 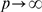


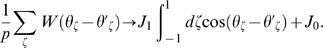
(17)


Introducing the definition of two uncorrelated maps (Eq. (1) with 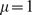) into Eq. (16), we can rewrite the angles in the intermediate maps as 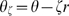, We can now integrate Eq. (17)
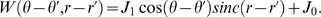
(18)Making use of the Euler formula for the 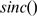 function
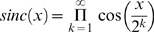
it is possible to derive
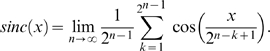
The first term of the infinite product in the Euler formula, or the first term in the limit sum, gives us 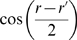. Comparing the coupling in Eq. (18), and the one derived for two maps, Eq. (2), we see that to first order, the synaptic coupling induced by the storage of the whole morph sequence, is equivalent to the storage of two correlated maps with 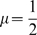.

**Figure 7:**
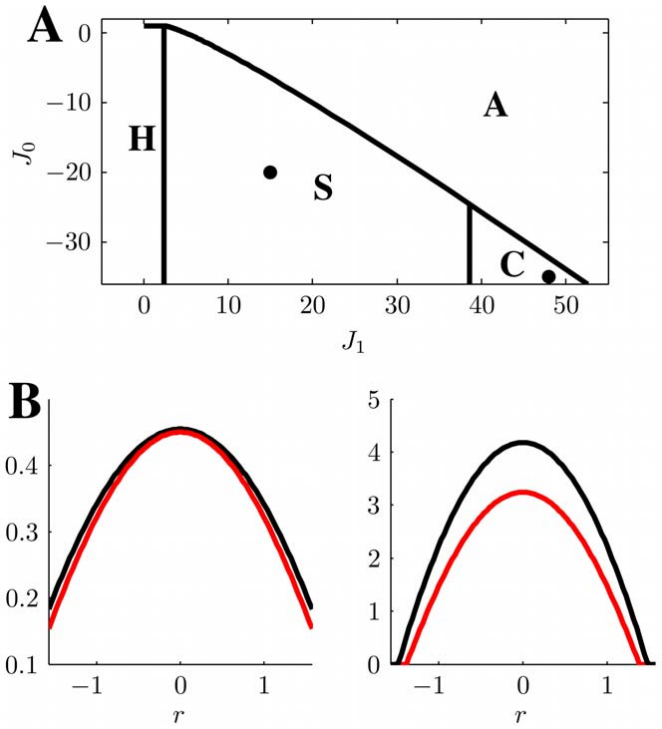
Comparison between whole morph sequence storage and two correlated maps with 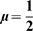. **A:** Phase diagram of the model storing two correlated maps with 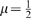, in the plane 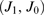. Note that with this value of the distance, the double ring regime is not achievable. (A) region correspond to amplitude instability. **B:** Slice of the steady activity along 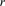, in correspondence of the maximum in 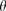. Left: parameters 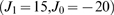 (dot in the 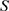 region). Right: 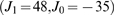 (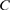 region); black curve, two stored maps; red curve, whole morph sequence stored. The bumps do not cover the whole range in both cases. External current 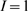.

**Figure 8:**
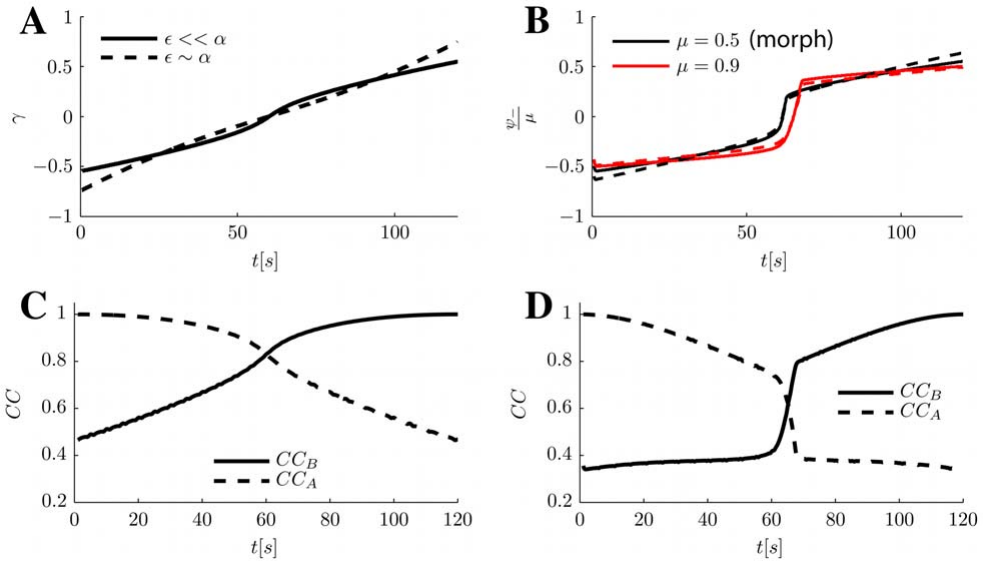
Representation switch along the morph sequence. Slow morph experiment between two reference environments, over 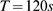 of simulation. The identity of the environment varies as 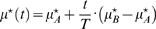 (linear change from environment 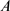 to 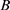), where 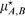 is either 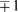 for the morph sequence, or 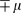 for two correlated environments. The input location is also time-varying, 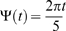 (a full circle in 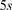). **A:** Double ring solution. Order parameter 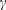 with time, both for weak 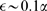 (solid curve) and strong external input 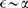 (dashed curve). The result does not depend strongly on the amplitude of the external input. Parameters 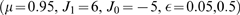. **B:** Cylinder regime. Order parameter 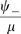 (location of the bump maximum in 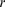) for the approximated storage of the morph sequence 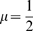 and for two correlated maps with 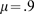, both for weak and strong input. Note how the sharpest transition, is exhibited by the weakly correlated maps. For the cylinder regime, the result is only mildly dependent on the strength of the external modulation. Parameters 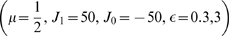 and 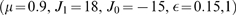. **C:** Instantaneous correlation coefficients between the double ring regime network activity during the morphing procedure, and network activity in environment 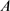 (dashed curve) or 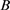 (black curve). Simulations with weak external input. **D:** As in C, for the cylinder solution. Note how the sharp transition is delayed compared to 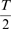.

In Fig. 7, we compare the network activity generated by the approximated coupling and the full result of Eq. 18, when the external input is constant. The results are qualitatively similar but the full morph case reaches the cylinder regime for lower 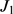 compared to the 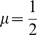 case. Note that the network storing the morph sequence shows the same dynamical pattern separation observed in the two maps case (Fig. 8), see next Section for a simulation example in a finite network with a finite number of encoded maps. The important difference, is that while the very correlations between maps forced the absence of neurons with certain labels, hence constraining the permissible region for a marginal solution in 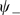, here the neurons cover the entire (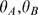) space. The result is purely due to the process of storing multiple maps along the morph sequence.

This morphing algorithm also yields a way of stimulating the network with positions in environments intermediate between 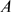 and 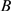 (with or without the intermediate maps encoded in the network). It is sufficient to use as a place specific input what we had in Eq. 13
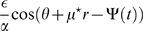
This time, the suitable range for the variable indexing the morph sequence 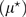 is the whole range 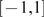, if using 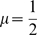 as an approximation for the morphed case, or the restricted 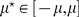 if the network is storing just two correlated maps. In the reference frame defined by the original coordinates (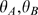), a change in the stimulated environment 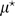 corresponds to a rotation of the axis representing the maximal external input; between a vertical axis (stimulus localized in environment 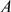, to an horizontal axis, stimulus localized in environment 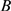.)

In the experiment of [32], the rat is trained until it develops two separate place coding for a single arena with different light configurations (representing two distinct environments). The advantage of this setup is that it allows, for instance, to slowly morph the light configuration between the two environments familiar to the rat. The experimental results shows a sharp transition around the middle of the light morphing (lasting 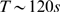) between the place representation in light configuration 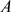 and 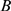. A link to these experimental results is provided by the use of time-varying external environment 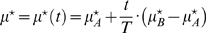, where 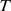 represents the duration of the morphing and 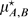 denote the upper and lower bounds of the range. An example usage of this protocol is shown in Fig. 8 for the approximated whole morph sequence storage, for two slightly correlated maps in the cylinder region of the parameter range and for the double ring regime. For each run we show the dynamics of the relevant order parameter for the regime under consideration, 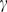 for the double ring case and 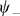 for the cylinder solution. In addition, we numerically solve the dynamics for a moving stimulus in either environment 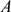 or 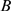. We use this as a reference for computing, at each time step, the correlation coefficient between the network activity during the morphing protocol and the activity in the fixed environment. The transition is sharpest for the storage of two slightly correlated maps. Note that similar results would be obtained by testing the network separately in each environment of the sequence (see e.g. [31]). The sharp transition is maintained when increasing the amplitude of the external tuned input, because a small tilt in the tuned input towards either map 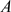 or 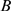 is sufficient to generate the dynamical pattern separation described in the previous Section. The transition in the cylinder regime occurs few seconds later than the one occurring in the double ring regime, which in turn happens in the middle of the morphing (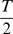). This delay is due to the time required for the bump to move from the region of 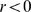 to 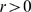, or viceversa (see also Fig. 5B). This result could be compared with the experimental results of [32]. The delay does not occur when testing the network in separate environments along the morph sequence.

There are two additional observations to be made (data not shown). The first one is related to the sharpness of the transition in the double ring regime; by further reducing the amplitude of the external input, the mean-field dynamics can produce a sharp transition between the environments representations, which is also delayed compared to the middle of the morphing period. The delay gets longer as the external input gets weaker, in extreme cases it happens just before the end of the morphing procedure. This sharp and delayed transition is not observed in microscopic simulations with up to 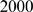 neurons, since the weak input is not able to overcome the local inhomogeneities in which the bump is trapped (see e.g. [18]). It is possible that in larger networks the transition can be observed. The fine-tuning of the external input strength required to have the transition around the middle of the sequence, makes the double ring regime a weaker candidate explanation for the experimental results of [32] compared to the cylinder regime.

The second observation concerns the dependency of the transition parameters on the velocity of the moving external input. We have noticed that the transition becomes smoother and closer to the middle as the velocity of the simulated animal is reduced. The details of the transition in a realistic setting would depend on the velocity history of the animal.

### Comparison with simulations

In order to verify that the results obtained in the previous Sections are not artifacts coming from our assumptions of having an infinite number of neurons (and maps, referred to the morphing procedure) we compare some of the MF predictions to simulations of networks with a finite number 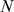 of neurons. Each neuron is assigned a random pair of labels (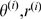, for the 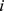-th neuron), from which we create either two maps with distance 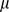, or a finite number 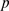 of maps (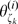, for the 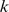-th map) along the morph sequence between two uncorrelated references (see *Methods* *- Numerical Methods*).

**Figure 9:**
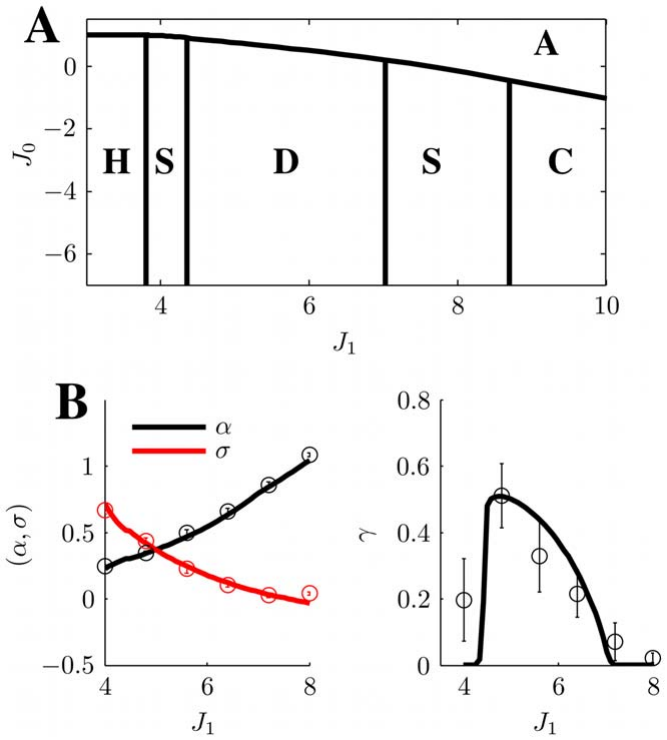
Comparison between MF solutions and microscopic network dynamics. **A:** Phase diagram from MF analysis for 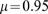. **B:** Order parameters from the numerical solution of MF equations (solid curves) with varying 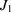; estimated order parameters from simulation results; dots with error bars, average and s.d. over 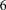 simulations with different realizations of the neural maps. 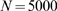 neurons. 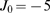.

In Fig. 9 we compare the order parameters from MF and estimated from simulations, at a fixed value of the distance between the maps and inhibition. Varying 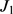, the solution goes through the double ring, single ring and cylinder regime. The order parameter 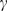 is particularly sensitive to the finite size of the network (and the randomized maps, see [18]).

**Figure 10:**
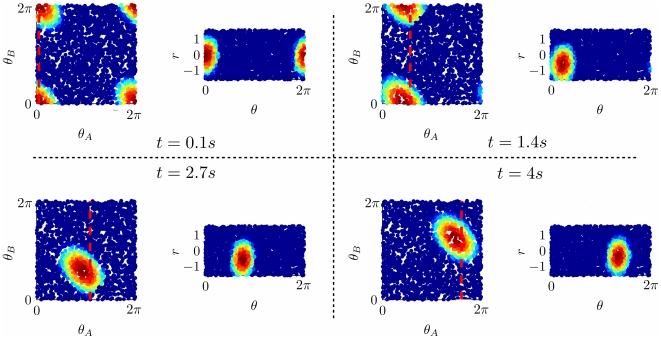
Dynamical pattern separation for a network storing a finite number of maps along the morph sequence. Sequence of snapshots of the network activity, in 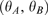 and 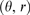 coordinates. The network with 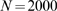 neurons is storing 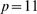 maps, equally spaced along the morph sequence. The external input is localized in map 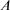 and evolves as 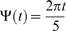. Each dot represent a neuron, with color coded activity. The dashed red line represents the maximum of the external tuned input. As the activity evolves in time from a random configuration, the subset of active neurons moves towards the negative 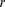 region and stays there moving only along 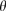. The parameters are 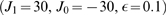.

Fig. 10 shows the time evolution of a network storing few maps from a morph sequence. This is the best example to show dynamical pattern separation at finite size, since it is less intuitive than the case of two correlated maps. From an arbitrary initial position, the bump of activity starts moving first towards negative 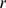 (increasing angles in map 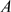), then along increasing 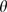 without changing its location in 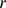. Note that, despite the presence of neurons everywhere in the (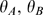) plane, the bump moves along an invisible barrier resulting from the storage of the morph sequence.

We have also verified that all the qualitative behaviors, number and type of solutions, unreliable coding, dynamical pattern separation and memory effect, are maintained when moving from maps on rings, to segments (either two correlated maps or morphed), as studied e.g. in [18], [37] for the single map (data not shown). Instead of having neurons arranged on a cylinder in the 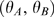 coordinates, as for the ring case (see e.g. Fig. 2**B,II**), the geometry resulting from two correlated linear maps would be an infinite strip. A strong enough map-specific interaction would produce a bump localized in both maps. An external moving input in one of the maps would move the bump on the strip up to the boundary, and then the bump would crawl along such boundary. Depending on the direction of the moving input or the identity of the stimulated environment, the bump can settle either in “upper” of “lower” part of the strip as in the cylinder regime.

## Discussion

We have studied a continuous attractor network model storing a pair of correlated maps. The storage of a morph sequence between two uncorrelated maps falls in this class of model, since it is approximately equivalent to the storage of two strongly correlated maps. The other relevant parameter for describing the possible network behaviors, beside the correlation between the maps, is the strength of map-specific interaction between neurons.

The analysis of the solutions of the system with a weak tuned external input, reveals several interesting behaviors. When the correlation between the maps is weak, neurons have two different tuning curves corresponding to the stimulus presentation in different maps. The representation is reliable, in that the single neuron response is consistent between presentations. This is the operating regime which is usually considered useful in place coding applications.

For higher correlations between the maps and weak map-specific interactions, each neuron possesses only one tuning curve, irrespectively of the stimulated map. In contrast to the previous regime, this one is rendered useless by the inability to represent fully the state of the external world, i.e. the identity of the environment in the context of place coding analogy.

We find another, novel regime for strong interactions and for *any* amount of correlation between maps. The surprising aspect of this regime is that the state of the world does not uniquely determine the state of the network; there is an additional degree of freedom in the network representation.

To a closer look, this additional freedom found in the novel regime is rich of consequences. When the external input location is randomly varied between presentations in one map, we can define the response of a neuron to a particular location as an average of the neuron activity over external input presentations in that location. In this context each neuron has different tuning curves relative to the different maps used in the stimulation, but the price to pay is unreliable coding; a neuron which should be active during a particular state of the world, could remain silent.

When the location of the external input changes smoothly in time on one map, some neurons develop a selectivity to the direction of change. When the increase happens on the other map, another subset of neurons fires. The overlap between the two subsets may be arbitrarily small, depending on the parameters choice. Neurons active in both maps would have tuning curves around similar values of the external input location. We refer to this phenomenon as dynamical pattern separation. There is an ambiguity in the network representation, due to the fact that the subset of neurons activating with the increase of the external location in map 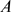, will also activate with a decrease of the location in map 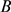. There are three possible experimental contexts in which this ambiguity does not arise.

A simple experimental context would arise if the input is tuned in only one of the two maps and the only parameter changing is the location of the external input. Given some state variable, like size and orientation of objects, or frequency of sound waves for instance, our model would produce respectively tuning for expansion/contraction, CW/CCW rotation and upward/downward frequency sweeps (all experimentally observed, see e.g. [40], [41]).

Our model provides a unique way for producing selectivity for the *direction of change* of a state variable, given a selectivity for the variable itself. Both kind of responses give rise to another interesting phenomenon: The current representation of the state of the world is influenced by the preceding one, even with an intervening delay. It is possible to read out from the network the direction of change of the state variable. This property may be exploited when solving delayed discrimination tasks (see [42] for data analysis and modeling in terms of remapping for a somatosensory discrimination task).

A second experimental context is related to place coding; the two environments should be considered as two distinct circular arenas which can be traveled only in one direction. Experimental observations show that when an animal is exposed to two environments, the majority of place cells have a place field in only one of the two environments (see e.g. [43], [44]). A possible experiment to test the model would consist in training the animals in two well differentiated environments. After measuring the distance between preferred locations for neurons having tuning curves (place fields) in both environments, one could train the animals in intermediate environments, which would correspond to the storage of the morph sequence in the model. For the novel regime of the model, the disappearance of the place fields in one of the environment would be predicted for neurons with very different preferred locations, and the remaining fields will converge to a common representation. Alternatively the training could be performed by using the initial two environments, and then slowly changing them across several training days to increase their similarity. This would correspond to the storage of two correlated environments.

A third experimental context is related to direction selectivity in place cells. Animals trained to shuttle back and forth in a one-dimensional track (a segment or a circle), have place cells showing selectivity to the direction of motion. For instance a cell could be active in a certain region of the circular environment when the animal is moving clockwise, while being completely silent when the animal moves counterclockwise. The link with our model is provided by the simple observation that the same 1D track, but walked in opposite directions, correspond to two different environments. Dynamical pattern separation would produce directional selective neurons, while a neuron having place fields in both environments would have similar preferred locations. In [45], place cells recorded from rats trained in a circular environment indeed showed bi-directional place fields in similar locations. There was however a systematic bias in the difference between the preferred locations in the CW and CCW directions of the majority of the bi-directional cells: place fields were displaced backward with respect to the direction of motion of the animal. We believe that this result, termed by the authors “prospective misalignment”, could be obtained in the context of our model in more than one way. One possibility is the introduction of an asymmetry in the synaptic connections (following [46]), with the asymmetry determined by the emerging direction selectivity of the neurons. The spread of activity due to the asymmetry would activate neurons earlier compared to the symmetric case, reproducing the prospective misalignment. A similar result could be obtained with short-term synaptic plasticity, which is known to produce a moving bump of activity ([47]). A third option could be the introduction of a systematic shift between the maps, possibly resulting from Hebbian learning of the configurations generated by the suggested asymmetry mechanisms.

In the experiments of [32], two environments correspond to two different light configurations in the same arena. A slow linear morph between light configurations results in a sharp transition from the population representation for one environment to the other. This is a promising experimental technique which is able to probe with unprecedented flexibility the dynamics of remapping between two environments or along a morph sequence [32], and could serve as a fertile ground for our model's predictions, hence for testing the attractor hypothesis. We show that, in agreement with the experiment, the slow morph protocol produces sharp transitions due to dynamical pattern separation. This result is even more significant considering the acknowledged difficulties in reproducing sharp transitions between correlated maps in a “traditional” setting [36]. The model predicts a transition between representations slightly delayed compared to half of the morphing period; it remains to be seen whether this occurs also in the experiment.

Our results can be related to experimental observations about changes in place representation between distinct environments. Two major classes of remapping have been observed when an animal is tested in two distinct environments: rate remapping, in which cells maintain the positions of their firing fields while differentially changing their amplitudes, and global remapping, where changes in firing location are observed in addition to firing rate modifications (see e.g. [43]). Based on these properties, we could associate the double ring regime to the global remapping and the cylinder regime to the rate remapping.

The model results can also be compared to experiments with sequences of continuously morphed environments. When animals explored intermediate environments, both sharp and smooth transitions in representations were observed in different experiments (see [31] and [30] correspondingly). Our model exhibits both sharp transitions between the place representations corresponding to intermediate environments (cylinder regime) and smooth transitions (double ring regime).

The linkage of cylinder and double ring regimes to sharp and smooth transitions respectively, taken together with the above mentioned association between these two model regimes with global and rate remapping, would be against the hypothesis made in [30] that related global remapping and sharp transitions on one hand, and rate remapping with smooth transitions on the other. In the present form, our model cannot be made compatible with this hypothesis. Since both the recordings of [31] and [30] contained populations of neurons exhibiting different transition behaviors, we speculate that the introduction of an additional selectivity for the environments (see below) could help in resolving the contradiction. Rate remapping would then correspond to a mixed single ring-cylinder regime (different subsets of the network would exhibit the different regimes), while global remapping would resemble a mix of the double ring and cylinder regimes.

A future extension of the model would include neurons with some form of selectivity for the context; each neuron would then be characterized not only by its location on the two maps, but also by selectivity indexes measuring its “preference” for the maps (e.g. [17]). This more realistic setting including selectivity would produce silent neurons and place fields with variable peak rates/widths even when storing a single map.

A second issue to be addressed is how the network can learn the synaptic structure from its inputs. The long-term plasticity (e.g. [33], [34]), could bring the network through various operating regimes depending on the training protocol. This could impose additional constraints on the model and provide additional predictions.

Finally, with the introduction of short-term plasticity [48]–[53], the network could exhibit an even richer repertoire of dynamics. This extension of the model would be an important step towards the experimental results of [32]. In this study, it was observed that when there is a fast switch between the two light configurations, the population vector sometime oscillates between the place representation of the environments, before settling on the current one. Preliminary results coming from the introduction of short term facilitation and depression in a network exhibiting a double ring solution, show that is indeed possible to observe oscillations between place representations. A detailed analysis of this behavior will be matter for a future report.

## Methods

### Numerical methods

To solve numerically the MF dynamics described by Eq. 5, we discretized 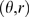 on 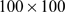 regular grid in 
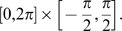
 The integrals in the rhs of the equations were estimated using a trapezoidal method. The system of ODEs were integrated with an adaptive 4-th order Runge Kutta scheme.

The simulation of the microscopic networks, whose results are reported in Figs. (9,10), were performed by solving numerically the system of ODEs
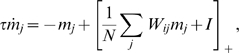
(19)where 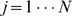 indexes the neurons. The matrix 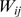 is built by summing the single map encoding 
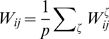
, where
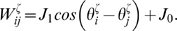
To obtain the 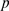 labels 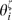 characterizing each neuron, we first randomly generated a 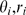 and used Eq. 1 for 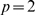 or Eq. 16 for 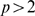. For the comparison of the simulation with the MF results in Fig. 9, we estimated from the steady state activity (compare with Eq. 22)
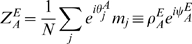
(20)

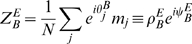


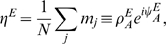
from which we constructed the estimates for the order parameters, using Eq. 24.

For the noisy simulations shown in Fig. 5**B**, we used a current-based version of the dynamics described by Eqs. 19:
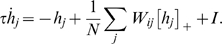
We then estimated the order parameters via Eqs. 20, using the firing rates 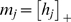. The noise was introduced as an additional term in the current
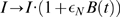
where 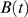 is a zero average, unit variance Gaussian 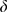-correlated noise. We used 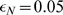 for the results in Fig. 5. The numerical solution was obtained using the Euler-Maruyama integration scheme.

The simulations performed in Fig. 7, for a network storing the whole morph sequence, were carried out as follows. Substituting the synaptic coupling obtained in Eq. 18 with the one in Eq. 2, it is possible to derive a dynamics for the “order function”
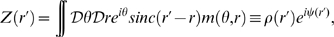
following the same procedure of *Methods* *- Reduced dynamics*. An order parameter 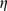 is defined exactly as in Eq. 22. The steady state activity of such dynamics
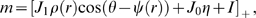
was compared with Eq. 4, for 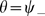 and 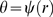 (in absence of a spatially tuned input 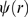 is constant).

The time constant 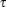 was set to 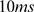 everywhere.

### Inverse transformation

The inverse transformation 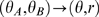 can be obtained from Eq. 1, defining
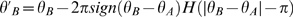
(21)

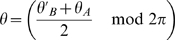


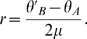
The 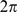 rotation in the first equation is just needed to select the shortest distance between two maps on a ring, and it is transparent for the connectivity given its periodicity. This rotation was implicitly assumed when defining the neurons locations along the morph sequence, Eq. 16.

### Reduced dynamics

A first reduction of the dynamics described by Eq. 2 is done using the first two Fourier components of the activity 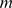 with respect to the two correlated maps 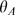 and 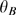, rewritten in terms of center map 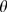 and the distance 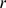 using Eq. 1. In line with [37] we define the following variables
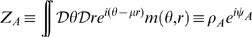
(22)

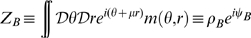


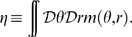
The variable 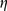 is just the average activity, while 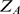 and 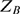 measure the spatial modulation of the network activity, in the map 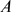 and 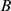 respectively. Intuitively their values tell us which angle of which map is instantaneously represented by the network.

The dynamics of the network activity 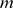, and of the order parameters 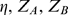, becomes
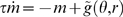
(23)

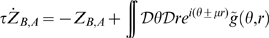


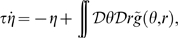
with
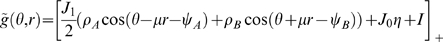
It is convenient to introduce dimensionless combinations of the order parameters to better expose the structure of the solutions, and then derive the dynamics of these new order parameters. From the two complex variables and the real one, we construct five new variables
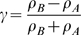
(24)

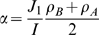


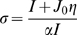


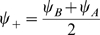


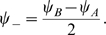
From Eqs. 23, after some algebra, it is finally possible to obtain the dynamics of the new order parameters, Eq. 5.

### Solutions properties

In *Methods* *- Phase diagram of the model*, we mentioned that the equation 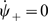 from Eqs. 5, i.e.
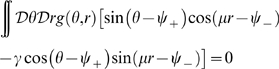
is automatically satisfied once the solution for the other four order parameters has been found. This can be seen using the fact that, by definition, the imaginary part of the real numbers 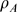 and 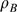 is 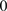. Since at steady state 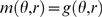, and 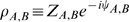 (Eqs. 22), by computing 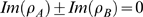 we can prove the property.

Another statement mentioned in *Methods* *- Phase diagram of the model*, is that fixed points solutions of Eqs. 5 with 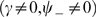 do not exist. Observing the shape of the network activity at steady state (Eq. 3) (setting the phase 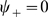 for convenience) which we rewrite here
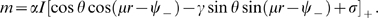
we would like to know, given the correlation between the stored maps 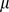 and the bump size 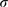, how much we can move the bump along 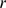 by increasing 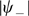 without having active neurons at 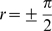. We first analyze the onset of the freedom of choice of 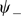, by requiring the bump to “fit” exactly the 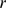 range; with a bigger bump, the only possible choice for 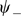 would be 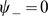, with a smaller bump it would be possible to move it along 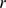. Hence, posing 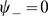, the activity at 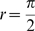 would be
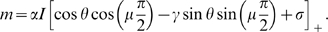
The angle 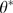 at which this activity is maximal is
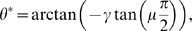
so the maximal activity at the 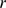 boundaries is
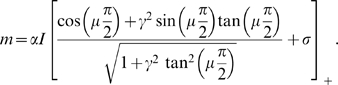
We recognize the first term inside the transfer function to be positive, so the only way to obtain a vanishing activity is to have 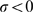. From Fig. 4 it is possible to see that the double ring solutions 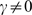 have always size 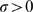.

In order to obtain the range of integration for 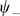 used to compute the average tuning curve in the cylinder regime (*Results* *- Tuned external input*), it is enough to consider the activity 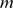 at its maximum in 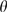


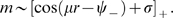
We want this bump in 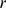 to at most touch the endpoints 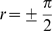. Given that the half-width of the bump is 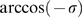, the allowed range for 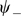 is 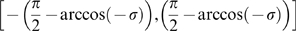.

### Stability

In order to study the stability of the homogeneous solution, corresponding to 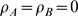 in Eq. 23 (i.e. 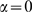 from 24), we can either linearize Eq. 23, or take a step back from the MF reduction which lead to Eq. 5, so to avoid division by 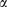. We take the second approach and redefine one of the order parameter, 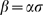. To study the stability of the solution, it is sufficient to look at the dynamics of 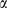 and 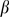. Posing 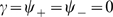, it is easy to verify that
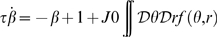


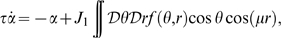
where the function 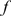 is defined as
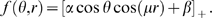
The matrix describing the linear dynamics for the vector of small perturbations 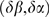 around the solution 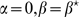 reads
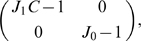
where
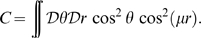
Therefore, two conditions must be satisfied for the solution to be stable: 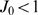 (amplitude instability) and 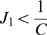. Evaluating the integral in 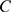 explicitly, we get the line of separation between the homogeneous solution and the localized bump (Turing instability), expressed in Eq. 7.

For the single, double ring and cylinder solution we can linearize directly Eq. 5, posing 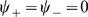. The matrix associated with the dynamics of the vector 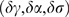, after using the fixed points equations (Eq. 8), is
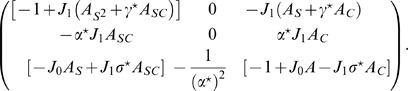
(25)We define
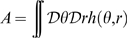
(26)

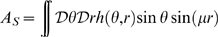


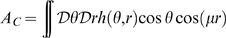


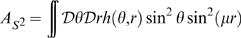


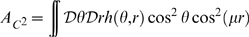


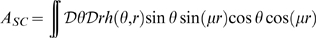
where
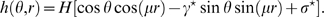
Using the identity 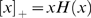, to write the function 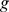 (Eq. 6), we see that
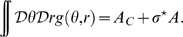
The fixed points equations can thus be rewritten in term of the quantities in Eq. 26
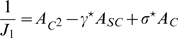
(27)

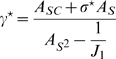


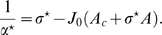



Let us examine the single ring and cylinder solution, 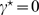. Given the symmetry in the integrand, 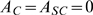 in this case. The stability matrix from Eq. 25 becomes then
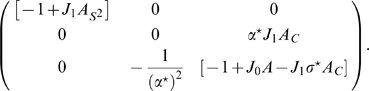
(28)


It is immediately seen that the eigenvalue corresponding to a destabilization of 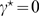 changes sign when 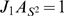. Substituting for 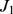 the expression in Eq. 27, it is easy to verify that this reproduces the curve of separation between the single and double ring regime described by Eq. 12. We analyze the remaining two eigenvalues by looking at the trace 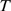 and the determinant 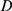 of the 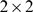 sub-matrix in the 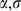 subspace (from 28):
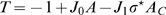


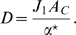
Given that 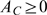, we see immediately that the eigenvalues have the same sign for 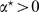, and one of them changes sign when 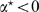. Recall that 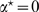 is the onset of amplitude instability we introduced without proof in Eq. 9. If we find that when 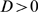 the trace is negative, then we know that 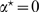 correspond to a destabilization of the solution.

Using Eq. 27, we see that imposing 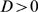 is equivalent to 
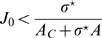
, and that the trace 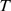 satisfies
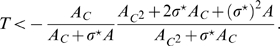
The numerator in the first term is non-negative (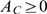). The denominator is simply 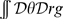, non-negative by definition. The denominator in the second term is 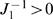, and we can write the numerator as
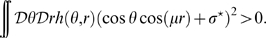
Finally, we numerically verified that the region of existence of the double ring solution coincides with its stability region.
